# Supplementary material for: Plasmodium falciparum Subtilisin-like Domain-Containing Protein (PfSDP), a Cross-Stage Antigen, Elicits Short-Lived Antibody Response Following Natural Infection with Plasmodium falciparum
Source: Cells. 2025 Jul 31;14(15):1184. doi: 10.3390/cells14151184 (PMC12346512; doi:10.3390/cells14151184)
Supplement: Supplementary file 1 [file cells-14-01184-s001.zip › cells-3596661-supplementary.pdf]

List of supplementary tables

Suppl Table 1: List and characteristics of selected peptides used for antibody production

Antibody	Target sequence	Range	Position	Protein length (aa)
α-PF3D7_1105800 peptide-1	KDSNRIIKGDSMTRC	31-45	N-terminal	266
α-PF3D7_115800 peptide 2	CKIDPEFKKIPNKNE	160-174	Middle	266
α-PF3D7_115800 peptide 3	CPLGKYQIEMPEFDK	94-108	Middle	266

Suppl table 2: Description of the study population.
SD: standard deviation; n: sample size, (%); percentage; mean age and parasitaemia were compared with the Kruskal Wallis test; gender, study site, episode frequency, clinical status, age group, parasite density group variables were compared with $\chi 2$ test.

		Site						
		**Accra**	**Ho**	**Kintampo**	**Navrongo**	**Adults**	**Total**	**P value**
**Gender** n(%)	Female	22(48.9)	72(46.5)	36(39.1)	52(49.1)	28(57.1)	210(47.0)	P<0.0001
	Male	23(51.1)	83(53.5)	56(60.9)	54(50.9)	21(42.9)	237(53.0)	P<0.0001
	**Total**	**45(10.1)**	**155(34.7)**	**92(20.6)**	**106(23.7)**	**49(11)**	**447(100)**	**P<0.0001**
	**P value**	0.85	0.21	0.00300	0.77	0.22	0.08	
**Age group** n(%)	2-6 years	12(26.7)	98(63.2)	37(40.2)	22(20.8)	0(0)	169(37.8)	P<0.0001
	7-15 years	33(73.3))	57(36.8)	55(59.8)	84(79.2)	0(0)	229(51.2)	P<0.0001
	Adults	0(0)	0(0)	0(0)	0(0)	49(100)	49(11.0)	
	**Total**	**45**	**155**	**92**	**106**	**49**	**447(100)**	
	**P value**	P<0.0001	P<0.0001	0.012	P<0.0001	--	P<0.0001	
	Mean ± SD [range]	**9.4 ± 3.2**[2-15]	**6.1± 2.9**[2-14]	**7.6± 2.9**[2-14]	**9.3± 3.1**[2-15]	**37.9± 16.1**[16-83]		P<0.0001
**Episode/yr** n(%)	0 time	8(17.8)	68(43.9)	4(4.3)	22(20.8)	14(28.6)	116(26.0)	P<0.0001
	1-2 times	37(82.2)	73(47.1)	40(43.5)	72(67.9)	34(69.4)	256(57.3)	P<0.0001
	≥3 times	0(0)	14(9.0)	48(52.2)	12(11.3)	1(2.0)	75(16.8)	P<0.0001
	**Total**	**45**	**155**	**92**	**106**	**49**	**447**	
	**P value**	P<0.0001	P<0.0001	P<0.0001	P<0.0001	0.0001	P<0.0001	
**Clinical status** n(%)	Asymptomatic	0(0)	0(0)	0(0)	56(52.8)	27(55.1)	83(18.6)	P<0.0001
	Uncomplicated	34(75.6)	155(100)	87(94.6)	50(47.2)	22(44.9)	348(77.9)	P<0.0001
	Severe	11(24.4)	0(0)	5(5.4)	0(0)	0(0)	16(3.6)	P<0.0001
	**Total**	**45**	**155**	**92**	**106**	**49**	**447**	
	**P value**	P<0.0001	---	P<0.0001	0.49000	0.42000	P<0.0001	
**Parasitaemia** n(%)	0-10000/µL	11(24.4)	89(57.4)	40(43.5)	81(76.4)	44(89.8)	265(59.3)	P<0.0001
	0-10000/µL	34(75.6)	66(42.6)	52(56.5)	25(23.6)	5(10.2)	182(40.7)	P<0.0001
	**Total**	**45**	**155**	**92**	**106**	**49**	**447(100)**	
	**P value**	P<0.0001	0.012	0.1	P<0.0001	P<0.0001	P<0.0001	
	Mean ± SD [range]	17959.3 ± 7613.2 [111-256390]	13094.1 ± 3887.9 [182-218235]	18982.1 ± 16905.3 [44-779100]	2254.6 ± 4405.3 [40-65520]	590.6 ± 2585.6 [40-65520]		P<0.0001

**Collection**	Day 0	45	78	69	---	---	192	0.001
**Time point**	Day7	43	78	64	---	---	185	0.0005
n(%)	Day21	43	---	60	---	---	103	0.025
	**P value**	0.96	0.92	0.64				

List of supplementary figures

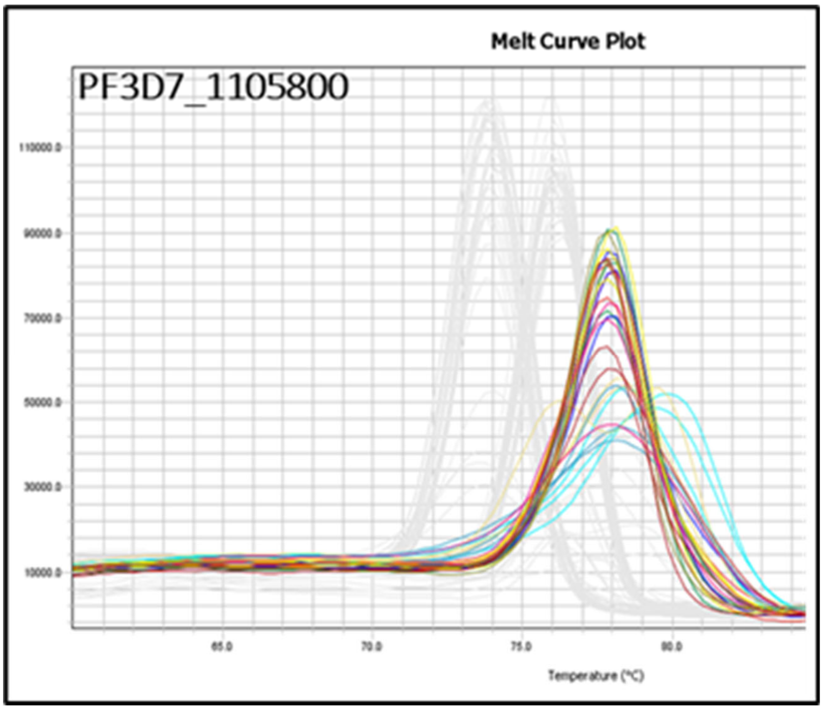

Suppl figure 1: Melt curves generated after RT-qPCR. The RT-qPCR was done using the Luna® Universal One-Step RT-qPCR Kit (New England Biolabs, Inc.) in triplicates following the manufacturer's instructions in a final volume of 10 µl. Experiments were performed on a QuantStudio 5 Real-Time PCR System (Applied Biosystems). A melt curve was performed on the final product to determine the specificity of the primers.

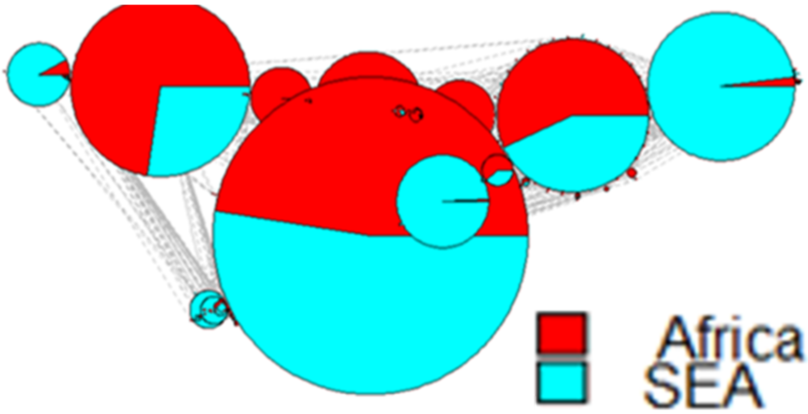

Suppl figure 2: Haplotype analysis for PF3D7_1105800 (*Pfsdp*) showing the major haplotypes present in both Africa and SEA. Gene fasta sequences were aligned using Mafft_auto and haplotype analysis was carried out using Pegas implemented in R environment.

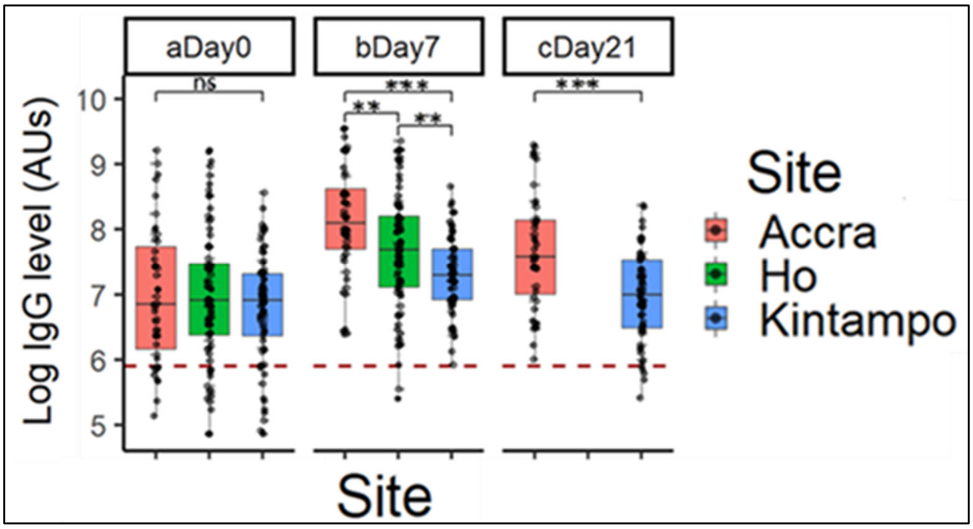

Suppl figure 3: Antibody level comparison between sites at different time points. Cut-off (-------) = mean ab level of the European plasma + 3SD. Mean IgG levels were compared using Kruskal Wallis (KW) and Mann-Whitney tests. When a KW test was significant (P-value < 0.05), a Dunnett's test was used as post-hoc for multiple comparisons. P-value code: ns: p > 0.05. *: p ≤ 0.05, **: p ≤ 0.01, ***: p ≤ 0.001.
